# A Recombinant Influenza A Virus Expressing Domain III of West Nile Virus Induces Protective Immune Responses against Influenza and West Nile Virus

**DOI:** 10.1371/journal.pone.0018995

**Published:** 2011-04-26

**Authors:** Byron E. E. Martina, Petra van den Doel, Penelope Koraka, Geert van Amerongen, Gunther Spohn, Bart L. Haagmans, Lisette B. V. Provacia, Albert D. M. E. Osterhaus, Guus F. Rimmelzwaan

**Affiliations:** 1 Department of Virology, Erasmus MC, Rotterdam, The Netherlands; 2 The National Institute of Public Health and the Environment, Bilthoven, The Netherlands; 3 Cytos Biotechnology AG, Zurich-Schlieren, Switzerland; The University of Chicago, United States of America

## Abstract

West Nile virus (WNV) continues to circulate in the USA and forms a threat to the rest of the Western hemisphere. Since methods for the treatment of WNV infections are not available, there is a need for the development of safe and effective vaccines. Here, we describe the construction of a recombinant influenza virus expressing domain III of the WNV glycoprotein E (Flu-NA-DIII) and its evaluation as a WNV vaccine candidate in a mouse model. FLU-NA-DIII-vaccinated mice were protected from severe body weight loss and mortality caused by WNV infection, whereas control mice succumbed to the infection. In addition, it was shown that one subcutaneous immunization with 10^5^ TCID_50_ Flu-NA-DIII provided 100% protection against challenge. Adoptive transfer experiments demonstrated that protection was mediated by antibodies and CD4+T cells. Furthermore, mice vaccinated with FLU-NA-DIII developed protective influenza virus-specific antibody titers. It was concluded that this vector system might be an attractive platform for the development of bivalent WNV-influenza vaccines.

## Introduction

West Nile Virus (WNV) belongs to the genus Flavivirus and is maintained in an enzootic cycle involving birds and mosquitoes, with humans and horses as “dead-end” hosts. WNV is circulating in the USA since 1999 and has infected more than 25,000 people with mortality rates of up to 2% [1]. Especially the elderly are at risk for developing severe disease and a poor outcome of infection, which may be attributed to an age-related decline of immune function [2], [3], [4]. Evidence is accumulating that the virus is moving southwards, putting millions of people in South-America and the Caribbean at risk [5], [6]. Several outbreaks of WNV infections in Europe indicate that the virus may also emerge in West European countries [7], [8], [9], [10]. Effective drugs for the treatment of WNV infections are not available and therefore safe and effective vaccines are needed to protect populations at risk. Several vaccine candidates have been tested in animal models [11], [12], [13], [14], [15], [16], [17], [18], [19], [20]. Most of these vaccine candidates are based on the glycoprotein E (gE), which is a target for the induction of virus-neutralizing antibody responses. In addition, the gE may be a target for T-cell responses [21]. The gE of flaviviruses consists of three domains (DI- DIII). DI and DII contain most of the cross-reactive B-cell epitopes and DIII most of the type-specific and neutralizing B-cell epitopes [16], [22], [23], [24]. Subunit vaccines based on DIII have been evaluated and proven effective in preventing severe infection in mouse models [11], [12]. However, high doses of recombinant DIII protein were needed to induce neutralizing antibody responses, indicating that DIII was poorly immunogenic. Elderly people that are at risk for severe WNV disease are also at risk for complications associated with influenza virus infections. Influenza viruses are an important cause of respiratory tract infections, affecting 5–10% of the human population annually with case-fatality rates of up to 1% [25], [26]. For the prevention of influenza and its complications, annual vaccination of high risk groups including patients with chronic disease, immune-compromised subjects and the elderly is recommended. Therefore, the availability of vaccines that could protect both against WNV and influenza virus infection would be desirable.

Here we describe the construction of a recombinant influenza virus vector that expresses DIII of the WNV gE protein. We hypothesized that the multimeric expression of DIII on recombinant influenza virus-infected cells or its presence on vector particles would increase its immunogenicity resulting in the induction of high titers of WNV neutralizing antibodies. Using influenza virus as a vector not only protective immunity was induced against WNV, but also against the vector. It was concluded that the use of recombinant influenza virus expressing WNV DIII is a promising approach that could afford protection against both viruses.

## Materials and Methods

### Cells

Madin-Darby canine kidney (MDCK; ATCC, CRL 1708) and Vero E6 cells were cultured in Eagle's minimal essential medium supplemented with non-essential amino acids, 100 IU/ml penicillin, 100 µg/ml streptomycin, 2 mM L-glutamine, 2% sodium bicarbonate, 1% HEPES, and 10% Fetal Bovine Serum (FBS) (all from BioWhittaker, Verviers, Belgium).

### Construction of DIII-expressing recombinant influenza virus

A recombinant attenuated influenza virus was constructed that expresses WNV DIII as a structural envelope protein. To this end, the region encoding DIII was amplified by RT-PCR using RNA extracted from WNV-NY99 infected Vero E6 cells. Subsequently, the 370 bp fragment was amplified using primers with respectively *EcoRI* and *SpeI* restriction sites (Fw: tggaattcATGGAACAACCTATGGCGTCT; Rev: gactagTCAATGATGATGATGATGATGGTCGA) and directionally cloned in frame with the N-terminal region of the influenza virus neuraminidase (NA), a type II membrane glycoprotein, essentially as described for recombinant influenza virus expressing green fluorescent protein (GFP) [27]. For generation of recombinant influenza viruses, bi-directional plasmids based on the gene segments of influenza virus strain A/PR/8/34 were used [28], [29]. The chimeric NA reverse-genetics plasmid was co-transfected with plasmids encoding PB1, PB2, PA, NP, HA, NS, and M1/M2 of influenza virus A/PR/8/34 [27], [28], [29], [30] into 293T cells and subsequently virus was rescued after passage in MDCK cells. Since this virus lacks a functional NA, virus was propagated in the presence of exogenous NA derived from V. cholera (Sigma). The virus was designated FLU-NA-DIII and a recombinant influenza virus expressing green fluorescent protein (FLU-NA-GFP) was used as negative control virus [27] in the experiments described below. To characterize FLU-NA-DIII, RT-PCR was performed with DIII- and influenza virus M1-specific primer sets (available on request). The identity of FLU-NA-DIII was further confirmed by sequence analyses of the PCR products.

### Characterization of recombinant virus

To confirm expression of recombinant protein, MDCK cells were infected with Flu-NA-DIII at an MOI of 0.01, without addition of exogenous NA. Cells were fixed with ice-cold methanol 20 hours later and incubated with mouse anti-influenza NP monoclonal antibody (ATCC, clone HB65), mouse anti-WNV polyclonal serum or a mouse anti-DIII monoclonal antibody (7H2; Bioreliance Corp., Rockville, USA). Horseradish peroxidase-labelled rabbit anti-mouse immunoglobulin (Dako, Glostrup, Denmark) was used as conjugate. The presence of specific antigens was demonstrated using 3-amino-9-ethylcarbazole (AEC, Sigma) as substrate according to the manufacturer's instructions. Alternatively, cells were harvested and lysed in buffer containing 150 mM NaCl, 1 g/L Nonidet-P40, 0,5 g/L sodiumdexycholate, 0,1 g/L SDS, 50 mM Tris.HCl. Proteins were separated by electrophoresis in 12% polyacryamide gel and subsequently transferred onto polyvinyldichloride difluoride Hybond-P membrane (Amersham Bioscience, UK). Membranes were incubated with either mouse anti-influenza NP monoclonal antibody or mouse anti-WNV polyclonal serum, and developed using a chemo-luminescence substrate according to manufacturer's instructions (ECL, Amersham).

### Vaccination-challenge experiments

In the first set of experiments, groups of six-weeks old C57BL/6 mice (n = 8 per group) were immunized twice either by the intra-nasal (i.n.) or the subcutaneous (s.c.) route using a dose of 10^6^ TCID_50_ of sucrose-gradient purified influenza viruses (FLU-NA-DIII or FLU-NA-GFP). Animals were immunized on day 0 and 14 and blood samples were collected on days 0, 14 and 42. Serum samples were tested for the presence of DIII-specific antibodies by ELISA and virus neutralization assay as described previously [12]. Neutralizing titers were expressed as the reciprocal of the highest serum dilution still giving 100% suppression of cytopathic effect. ELISA titers were expressed as the reciprocal of the highest serum dilution that resulted in an optical density higher than 0.200. Titers <50 were considered negative based on cutoff values established with sera from mice not infected with WNV. In addition, sera were tested for the presence of influenza virus specific antibodies. To this end, sera were treated with cholera filtrate and after heat inactivation serial two-fold dilutions were prepared. A hemagglutination inhibition (HI) assay was performed using a standard protocol with 1% turkey erythrocytes and 4 HA unit of influenza virus A/PR/8/34 as described [31]. All animal experiments were approved by the animal ethics committee of the Erasmus MC Rotterdam, The Netherlands.

IFN-γ ELISPOT was used as indicator for the induction of DIII-specific T-cell responses in the spleen. Plates were coated overnight with 1,5 µg/well of anti-IFN-γ antibody (AN18; Mabtech, Germany). Splenocytes were cultured in triplicates at a density of 2×10^5^ cells per well in a volume of 150 µl at 37°C and stimulated 10 µM DIII derived peptide 39f (VNPFVSVATANAKVL). After incubation for 48 hours at 37°C in 5% CO2, plates were washed thoroughly and incubated with 1 µg/ml biotin-conjugated anti-IFN-γ antibody (R4-6A2; Mabtech). Plates were developed by adding GABA-labeled-streptavidin (U-cyTech, Utrecht, Netherlands) according to the manufacturer's instructions, and spots were counted using an ELISPOT reader (Bioreader 3000, Bio-Sys GmbH).

To determine the dose-protection range, groups of mice (n = 8) were immunized twice (day 0 and 14) s.c with 10^1^, 10^2^, 10^3^, 10^4^, and 10^5^ TCID_50_ of sucrose-gradient purified FLU-NA-DIII or FLU-NA-GFP.

On day 42 post vaccination, all animals were infected subcutaneously with a lethal dose of WNV-NY99 (1×10^6^ TCID_50_). Mice in all groups were observed each day for illness, weight loss, and death for a period of 14 days.

### Passive transfer experiments

To assess which arm of the adaptive immune response was responsible for protection against challenge infection, adoptive transfer experiments were performed using serum, purified CD4+T cells and CD8+T cells obtained 4 weeks after the booster immunization with FLU-NA-DIII or FLU-NA-GFP. MACS magnetic beads (Miltenyi Biotec, Auburn, CA) were used to separate the CD4+ (L3T4) and CD8a+ (Ly-2) T-cell fractions from mouse splenocytes, as instructed by the manufacturer, which yielded purified CD3+CD4+ and CD3+CD8+T cell preparations with <0.5% contaminating cells. Lymphocytes were washed three times with, and resuspended in phosphate-buffered saline (PBS). Eight weeks old recipient mice (n = 5) received 200 µl immune serum, 5×10^5^ CD4+ or 5×10^5^ CD8+T cells by the intra-peritoneal route. Four hours post transfer the animals were infected by the s.c. route with 100 TCID_50_ of WNV-NY99, which is a lethal dose for mice of this age. Eight days after challenge, all mice were sacrificed and the virus titers in the brains were determined as described previously [12].

### Statistical analysis

Differences in Kaplan-Meier survival curves between the groups were assessed using the log-rank test. Data for serum antibody titers and viral titers in brains were analysed using the two-sided Student's *t* test. All statistical analyses were performed using GraphPad Prism version 4 software (Graphpad Software, San Diego, USA). Values of *P≤*0.05 were considered statistically significant.

## Results

### Vaccine Characterization

**Figure 1:**
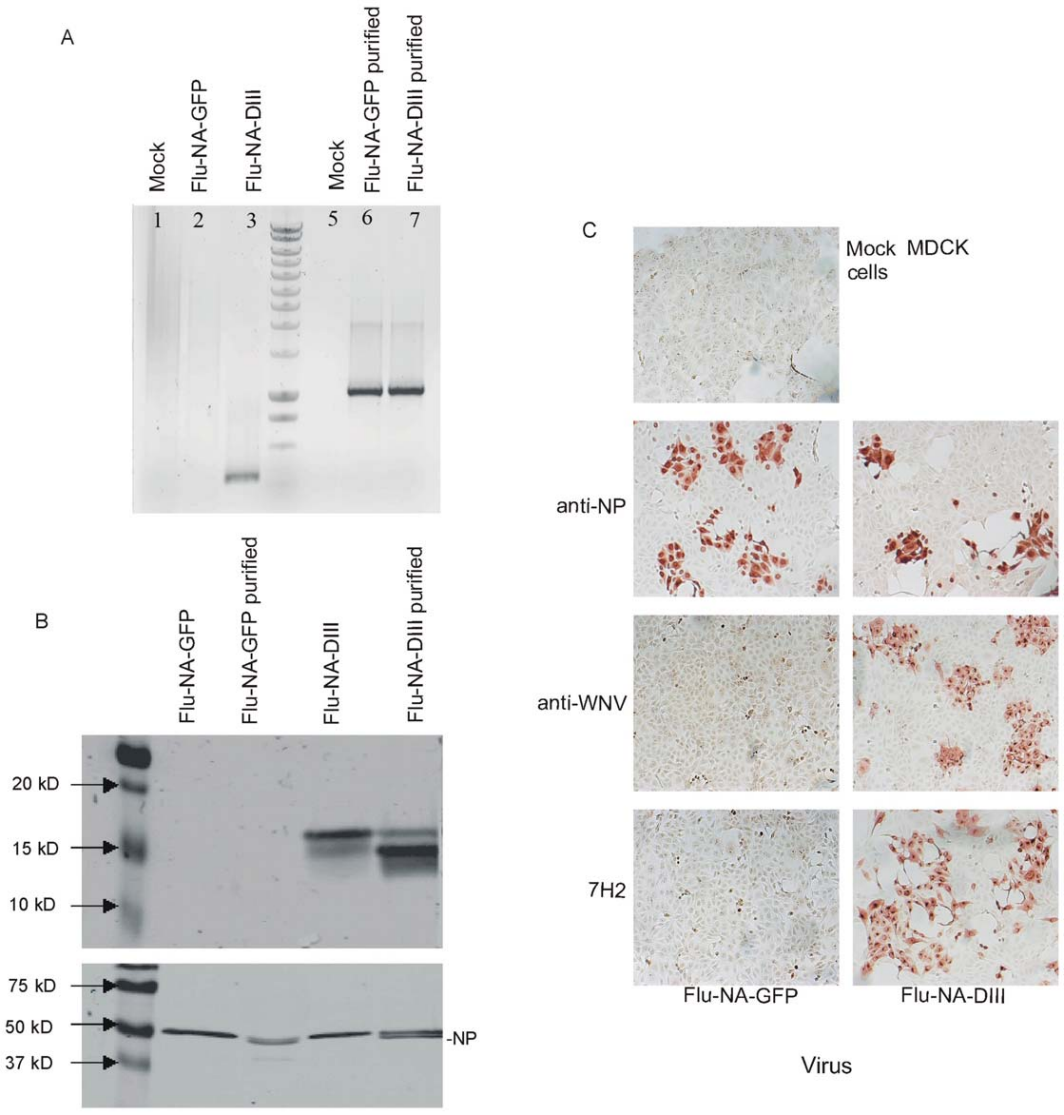
Characterization of recombinant influenza A viruses. RT-PCR analysis of Flu-NA-GFP and Flu-NA-DIII RNA extracted from MDCK cells 20 hours after infection (A). Amplicons were separated in 1% agarose gel. For amplification WNV-DIII (lanes 2 and 3) and influenza A (lanes 6 and 7) virus-specific primers were used. Expression of DIII was analyzed by Western blot analysis (B). Viral proteins in lysates of infected cells or in sucrose gradient purified virus preparations were separated by SDS-PAGE and transferred to PDVF membranes, which were incubated with a DIII specific monoclonal antibody 7H2 (upper panel) or an influenza virus NP specific monoclonal antibody (ATCC, clone HB65; lower panel). Expression of DIII was also confirmed by immuno-staining of MDCK cell infected with FLU-NA-GFP and FLU-NA-DIII (moi = 0.01) with NP- and DIII-specific antibodies as indicated (C).

As shown in figure 1A, with the influenza virus M1 primer set a signal of 1.1 kb was observed with the RNA extracted from both FLU-NA-DIII and FLU-NA-GFP virus stocks, while only the DIII primer set resulted in an amplicon of 451 bp with FLU-NA-DIII. Also nucleotide sequence analysis confirmed the identity of both viruses.

As expected, the recombinant influenza virus Flu-NA-DIII was unable to spread in culture due to lack of NA activity. The fact that no infectious virus could be recovered from MDCK cells infected with Flu-NA-DIII in presence of trypsin up to three days after infection, indicated that no wildtype influenza virus was present in the recombinant viral stock. Increase in Flu-NA-DIII titers was only observed *in vitro* after addition of exogenous NA in a dose-dependent way. The minimal concentration of exogenous NA needed for optimal propagation of NA deficient Flu-NA-DIII was 0.1 mIU/ml. Next, Western blot analysis performed with cell lysates of virus-infected MDCK cells and antibodies directed against influenza virus NP and WNV DIII, showed that both FLU-NA-DIII and FLU-NA-GFP expressed influenza virus NP, which was also detected in purified virus preparations (figure 1B). In contrast, the presence of the 15 kDa DIII was only observed in FLU-NA-DIII infected cell lysates and purified FLU-NA-DIII preparations. The presence of GFP in influenza virus FLU-NA-GFP infected cells and purified virus preparations was demonstrated previously [27]. The expression of DIII was also demonstrated in infected cells with an immunostaining technique using WNV-specific antibodies (Figure 1C). Thus, an attenuated NA-deficient recombinant influenza virus was constructed that expresses WNV-DIII and carries the DIII as a structural envelope protein.

### Immunogenicity and efficacy studies

**Figure 2:**
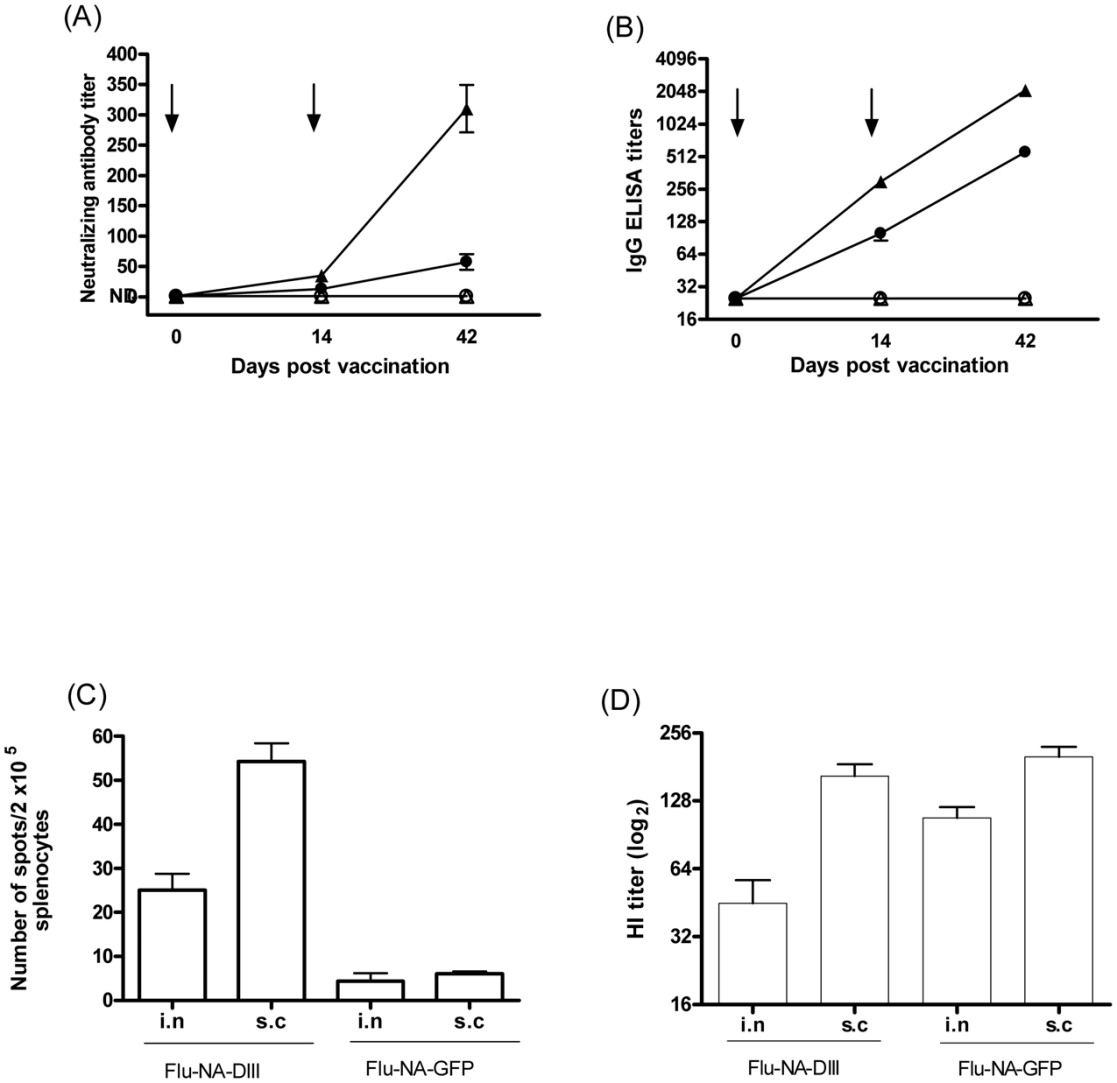
Induction of WNV-specific immune responses by vaccination with FLU-NA-DIII. WNV-neutralizing antibodies were detected by virus neutralization assay (A) and DIII-specific IgG antibodies by ELISA (B) in serum obtained from mice vaccinated with FLU-NA-DIII i.n. (•) or s.c. (▴) and FLU-NA-GFP i.n. (○) or s.c. (▵) at the indicated time points. Arrows indicate time points of vaccination. The data are expressed as average titers per group (n = 10) ± SD. Cellular DIII-specific responses were determined by IFN- γ ELISPOT assay (C). Splenocytes obtained on day 42 were stimulated with 10 µM peptide (VNPFVSVATANAKVL) and the numbers of cells producing IFN-γ per 2×10^5^ cells were determined in mice vaccinated with FLU-NA-DIII or FLU-NA-GFP as indicated. Each experiment was performed twice in triplicate. Results are indicated as mean ± standard deviation. Induction of Hemagglutination titers after immunization with FLU-NA-DIII and FLU-NA-GFP. Mice (n = 8) were immunized intranasally (i.n.) or subcutaneously (s.c.) with Flu-NA-DIII or FLU-NA-GFP (D).

As shown in figure 2, both i.n and s.c immunization with FLU-NA-DIII induced DIII-specific antibodies. The s.c route was more efficient than the i.n route both in terms of induction of virus-neutralizing titers (Figure 2A) and WNV-specific IgG ELISA titers (Figure 2B). On day 14 slightly higher neutralizing antibody titers (range: 20–80) were measured in mice vaccinated s.c with Flu-NA-DIII, compared to the mice that received the same vaccine i.n (range: 10–40). A clear booster response was seen in all animals that received the Flu-NA-DIII four weeks after second vaccination, resulting in statistically significant differences between groups that were vaccinated i.n and s.c (*P*<0.001). The use of FLU-NA-GFP did not induce DIII-specific antibodies. In addition, immunization with FLU-NA-DIII induced WNV DIII specific cellular immune responses as detected in an IFN-γ ELISPOT assay after stimulation of splenocytes with a DIII derived peptide (VNPFVSVATANAKVL) representing a T helper cell epitope (Figure 2C). Consistent with the antibody responses, the IFN-γ response was significantly higher in mice vaccinated s.c as compared to i.n. vaccinated animals (*P*<0.05). Four weeks after second vaccination, all mice developed antibody responses against the homologous influenza virus A/PR/8/34 in the HI assay. After two immunizations with either FLU-NA-DIII or FLU-NA-GFP, mice that received the vaccine by the i.n and the s.c routes developed detectable antibody titers (Figure 2D). Four weeks after the second immunization these animals had HI titers ranging from 40–160, with higher titers measured in the groups that received the vaccine subcutaneously.

**Figure 3:**
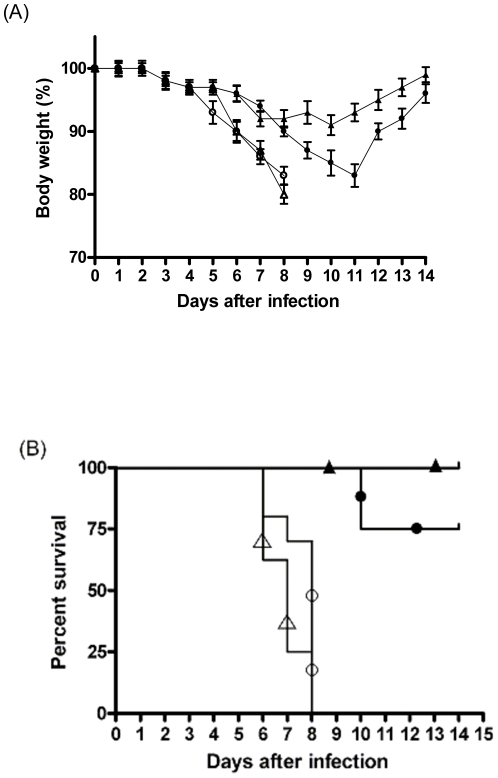
Loss of body weight after challenge-infection with WNV. Mice (n = 8) were vaccinated intranasally with Flu-NA-DIII (•) or Flu-NA-GFP (○) or by the subcutaneous route (▴ and ▵, respectively). The daily weights of each animal were calculated compared to their respective weight on the day of challenge, and data are shown as the average percentage of initial weight for each group. Error bars represent the standard error for all samples available at that time point. Subsequently, the mice were challenged subcutaneously with 10^6^ TCID_50_ WNV-NY99 and weighed daily. The mean body weight is expressed as the percentage of the body weight before challenge infection (A). The survival rates of mice after challenge infection with WNV-NY99 are depicted as Kaplan-Meier survival curves (B). The difference in survival rate between Flu-NA-DIII and Flu-NA-GFP vaccinated mice was statistically significant as determined by the logrank test. The symbols for the respective groups are the same as in panel A.

To assess the protective efficacy of the immune responses induced with FLU-NA-DIII and FLU-NA-GFP, groups of ten mice were challenged with a lethal dose of 10^6^ TCID of WNV-NY99 given subcutaneously. Mice vaccinated with FLU-NA-GFP developed clinical signs characterized by ruffled fur and hunched posture six days after infection onward, lost around 20% of their body weight eight days post infection, and all animals died or had to be taken out of the experiment within eight days post infection (Figure 3). Weight losses were first observed around day 6 post infection, which coincided with signs of paralysis. In contrast, mice vaccinated with FLU-NA-DIII lost body weight at a slower rate and eventually recovered from the infection with WNV-NY99. Especially subcutaneously vaccinated mice suffered less from the infection, lost weight minimally, and recovered quickly. Survival rates were 100% and 75% after s.c and i.n vaccination with FLU-NA-DIII, respectively. FLU-NA-GFP vaccinated mice were not protected from infection, which indicates that innate immune responses were not responsible for the observed protection.

**Figure 4:**
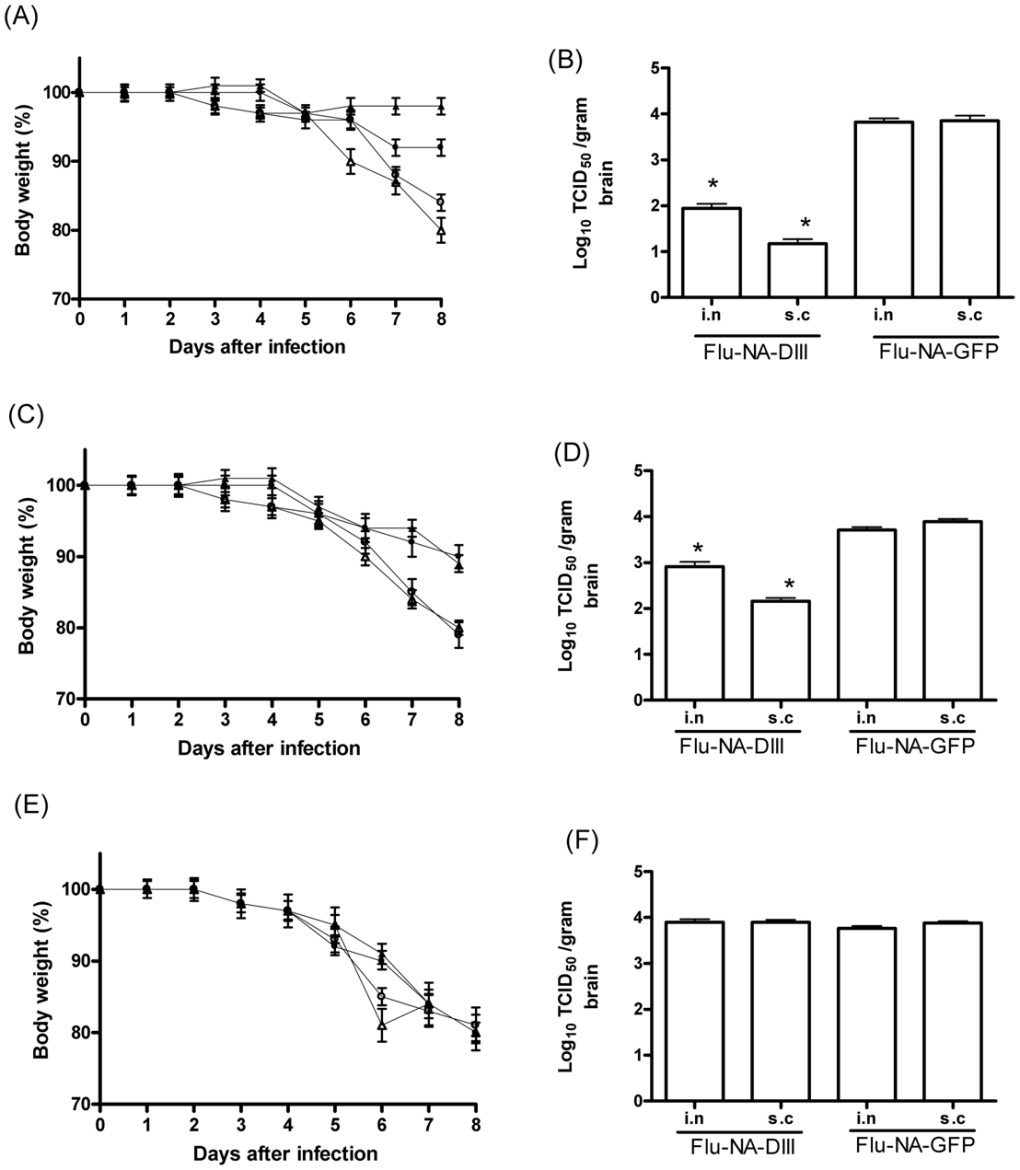
Protection against challenge infection is mediated by humoral and CD4+T cell responses. Recipient mice received serum (A and B) CD4+T cells (C and D) or CD8+T cells (E and F) obtained from mice that were vaccinated with FLU-NA-DIII (closed symbols) or FLU-NA-GFP (open symbols) by the i.n route (• and ○ respectively) or s.c. (▴ and ▵ respectively) and were subsequently infected with 100 TCID_50_ WNV-NY99. Loss of body weight (A,C and E) and virus titers in the brain were determined eight days post challenge infection (B, D and F). The results represent the mean values of groups of five mice. Error bars indicate the standard deviation; * indicates a statistically significant difference compared to control groups receiving serum or T cells from FLU-NA-GFP vaccinated mice (determined by t test). The daily weights of each animal were calculated compared to their respective weight on the day of challenge, and data are shown as the average percentage of initial weight for each group (A, C, E). Error bars represent the standard error for all samples available at that time point.

Adoptive transfer experiments were performed to study correlates of protection. Mice that received serum, CD4+ or CD8+T cells obtained from FLU-NA-GFP vaccinated mice lost up to 20% of their body weight and had mean brain virus titers of 10^3.8^ TCID_50_/gram tissue. In contrast, transfer of FLU-NA-DIII immune serum to naïve recipient mice reduced the weight loss and virus replication in the brain significantly (*P*<0.05; figure 4 A and B). A similar effect was observed after the transfer of CD4+T-cells obtained from FLU-NA-DIII vaccinated mice (*P*<0.05; Figure 4C and D), whereas the transfer of FLU-NA-DIII immune CD8+T cells did not confer any protection against disease caused by infection with WNV-NY99 (Figure 4E and F).

To determine the dose-range of FLU-NA-DIII able to induce protective immunity after two immunizations, groups of mice were immunized with a range of doses (10^1^–10^6^). Doses ranging from 10^1^–10^3^ did not protect animals against lethal infection (Table 1). Immunization with 10^4^ TCID_50_ of FLU-NA-DIII resulted in 75% (6/8) survival, while 10^5^ and 10^6^ TCID_50_ FLU-NA-DIII protected 100% mice against lethal infection. To investigate whether one immunization could offer protection, mice were immunized once with either 10^5^ or 10^6^ TCID_50_ FLU-NA-DIII. On day 42 mice were challenged WNV. Both doses resulted in 100% survival after one immunization, whereas animals that received FLU-NA-GFP were not protected.

**Table 1:** Proportion survival following one (1X) or two (2X) immunizations with FLU-NA-DIII or FLU-NA-GFP.

Vaccine dose(TCID_50_)	FLU-NA-DIII (2X)(survival/total)	FLU-NA-GFP (2X)(survival/total)	FLU-NA-DIII (1X)(survival/total)	FLU-NA-GFP (1X)(survival/total)
10^1^	0/8	0/8	ND	ND
10^2^	0/8	0/8	ND	ND
10^3^	0/8	0/8	ND	ND
10^4^	6/8	0/8	ND	ND
10^5^	8/8	0/8	7/8	0/8
10^6^	8/8	0/8	8/8	0/8

ND: Not done.

## Discussion

In the light of the continuing threat of WNV to the western hemisphere, the availability of a safe and effective vaccine is crucial. In the present study we have evaluated the immunogenicity and protective efficacy of a recombinant influenza virus expressing DIII of WNV in a mouse model. These data confirm that vaccine-induced humoral and CD4+T cell responses contribute to protective immunity against WNV challenge infection. The protective role of antibodies against WNV has been demonstrated in various studies [32], [33], [34]. Several epitopes have been defined on gE of WNV, involved in cell attachment, trimerization and fusion. The epitopes that induce potent neutralizing antibodies are located on the upper lateral surface of DIII, and these antibodies can block infection efficiently at a post-entry step [35], [36], [37].

In addition to DIII-specific antibodies, our results show that DIII-specific CD4+T-cells provided partial protection against neuro-invasive disease in mice, which is in concordance with previous studies showing the protective effects of gE-specific CD4+T cells [38], [39]. Here we show that CD4+T cell responses specific for the ectodomain of DIII, were sufficient to afford partial protection against WNV virus infection. The mechanism underlying the protective effect of these CD4+T-cells is unclear, but levels of IFN-γ production inversely correlated with virus titers in the brain. Several studies have shown that CD8+T cells play a role in elimination of WNV from infected tissues and protection against lethal disease [40], [41], [42], [43]. Adoptively transferred CD8+T cells obtained from FLU-NA-DIII vaccinated mice failed to confer protection in the present study, which may be attributed to the absence of CTL epitopes in DIII recognized by C57BL/6 mice.

Attenuated recombinant influenza viruses have been proposed as attractive vaccine vectors, since they are highly immunogenic in the absence of virulence [44], [45], [46], [47], [48]. Also NA-deficient influenza viruses have been suggested as safe and potent vector vaccine candidates [48], [49]. An additional advantage of using NA deficient influenza A virus is that it can be used as a safe and effective vaccine against seasonal influenza. Both the FLU-NA-GFP and FLU-NA-DIII recombinant viruses induced antibody responses against influenza virus A/PR/8/34. Serum HI titers of ≥40 are considered to be protective against influenza [50], and vaccination with the NA deficient virus met these minimal requirements. Therefore it is anticipated that the use of these attenuated influenza viruses will afford protection against infection with homologous viruses.

Several vectored vaccines have been developed against WNV [19], [51], [52], [53], [54], [55], most of them tested as candidate vaccines for human use. In most of these studies the vectored vaccines expressed the secreted form of WNV glycoprotein E, which were administered twice either via the intraperitoneal or the subcutaneous route and resulted in 80–100% efficacy. One study describes the administration of a recombinant vesicular stomatitis virus vaccine via the intranasal route, which resulted in 80% survival rates [54], in line with the data presented in this manuscript. Our study is the first to describe the use of domain III of WNV glycoprotein E, with efficacy rates similar to vectored vaccines expressing the complete glycoprotein E of WNV.

In this study, the use of recombinant influenza virus as vector for the delivery of WNV DIII resulted in WNV-specific immune responses and HI titers greater than 40. Subcutaneous immunization was more efficient than intranasal administration of the vaccine, which was included as well since influenza viruses typically target the respiratory tract. Apparently, the route of administration affected the outcome of the immune responses and the protective potential of this candidate vaccine, which was demonstrated also for other vaccine preparations [56], [57], [58]. The influenza virus specific antibody levels that were induced in all vaccinated mice indicated that these animals were protected against infection with influenza A/PR/8/34 as well.

Collectively, the data presented in the present study indicate that an attenuated NA-deficient recombinant influenza virus is a promising bivalent vector-vaccine candidate for the induction of DIII-and HA-specific antibodies as well as DIII-specific T cells. Especially subcutaneous administration of FLU-NA-DIII induced strong immune responses and afforded substantial protection against infection with WNV-NY99 in mice. Further studies are ongoing to determine how soon after immunization the vaccine is effective and the level and longevity of protection provided by a single immunization. Further evaluation of this vector system as a WNV and influenza A virus bi-valent vaccine seems warranted.
